# Pathways to Real World Evidence From the Real World of School‐Based Physical Fitness Testing (SB‐PFT)

**DOI:** 10.1002/lrh2.70108

**Published:** 2026-07-26

**Authors:** Anita Walden, Allen Yiu, Gregory Welk, Yawen Guo, Shlomit Radom‐Aizik, Margaret Schneider, Allyson Reeds, AbdulMalik Shakir, Dan M. Cooper

**Affiliations:** ^1^ Department of Genetics University of North Carolina School of Medicine Chapel Hill North Carolina USA; ^2^ General and Community Pediatrics Children's National Medical Center Washington DC USA; ^3^ Department of Kinesiology Iowa State University Ames Iowa USA; ^4^ Bren School of Information and Computer Sciences, University of California at Irvine Irvine California USA; ^5^ Department of Pediatrics Pediatric Exercise and Genomics Research Center, School of Medicine, University of California at Irvine Irvine California USA; ^6^ Institute for Clinical and Translational Science, School of Public Health, University of California at Irvine Irvine California USA; ^7^ Orange County Department of Education Orange County California USA; ^8^ Shakir Consulting LaVerne California USA; ^9^ Department of Pediatrics School of Medicine, Institute for Clinical and Translational Science, University of California at Irvine Irvine California USA

**Keywords:** domain analysis modeling, real world data, real world evidence, school‐based fitness testing

## Abstract

**Introduction:**

Unique challenges of data quality, standardization, and interoperability face non‐traditional sources of real world data (RWD), those outside of the common RWD source, the electronic health record (EHR). The experience of our clinical, research, and community group in addressing the endangered school‐based physical fitness testing (SB‐PFT) data set may be useful as a paradigm for a variety of nontraditional instances of health‐related RWD. Until recently, SB‐PFT was mandated annually within public schools in ~60% of U.S. children and adolescents representing one of the most robust and inclusive data sets available in youth. When implemented rigorously, SB‐PFT produced data that improved pediatric health and student learning. Driven by perceived and real gaps in data quality, inconsistent and insensitive school‐site implementation, and uneven evidence for health‐relevance, calls for revamping (and, in some cases, ending) school testing are growing.

**Methods:**

To preserve and enhance this unique resource, SB‐PFT must incorporate emerging science and technology. We describe here the rationale, structure, and process involved in our building an HL7‐associated Domain Analysis Model for SB‐PFT. Our team consisted of clinicians, exercise scientists, school personnel, publish health officials, parents and students. The structure of the DAM was facilitated by data standard experts and skilled Unified Modeling Language professionals. Administrative support was rendered by our institution's NIH‐funded Clinical Translational Science Award.

**Results:**

The DAM will serve as a necessary first step in integrating Fast Healthcare Interoperability Resources (FHIR) and Learning Health Systems (LHS) models essential to realize the full potential of SB‐PFT data in advancing child health.

**Conclusion:**

The experience presented here can serve as a roadmap for addressing critical issues of data standardization and RWE generation in nontraditional instances of RWD. The DAM can be deployed in SB‐PFT data exchange in real world settings.

## 1 Overview

In this Experience Report, we review our ongoing approaches to improve the quality and interoperability of a unique and remarkably inclusive instance of health relevant real‐world data (RWD), school‐based physical fitness testing (SB‐PFT). We outline the elements, reasoning, and process that led to our decision to implement a Domain Analysis Model (DAM) in conjunction with the international standards organization HL7 focused on school‐site fitness testing. HL7 defines a DAM as, “A representation of the static and/or dynamic semantics of a subject‐area‐of‐interest (i.e., domain) in a manner that enables harmonization of the various perspectives of the stakeholders in the domain while also providing the foundations required to create logical platform‐independent and implementation platform‐dependent models of information artifacts and/or applications whose semantics involve concepts from the domain.” [1] In short, a DAM is a structured approach to incorporating multiple stakeholder perspectives into a formal definition of a domain (in this case, school‐based physical fitness testing), and is a necessary step to include RWD in the electronic health record.

We present the composition of the stakeholders who invested substantial time and thought into the DAM process, the successes and challenges that accompanied the process, and the more generalizable lessons learned for improving RWE from nontraditional sources. We suggest the steps necessary to build on the DAM and implement the Learning Health System (LHS) model which is specifically designed to align science, informatics and culture to promote continuous improvement and innovation in health care delivery [2, 3]. While the DAM itself does not inevitably create an LHS, the identification of critical data and terminology relevant to a particular health problem can inform the eventual development of a successful LHS. The vision for a future SB‐PFT LHS would focus on youth physical fitness and physical literacy, and might serve as a more generalizable model in which the DAM is a step in formulating an LHS. A long‐term goal of this effort is to streamline the inclusion of school‐site fitness testing into the electronic health record (EHR), providing health care providers with a cost‐effective and actionable critical biomarker of pediatric health.

The exponential increase in the use of RWD [4, 5, 6, 7] (traditionally defined as data relating to patient health status and collected from various sources such as EHRs, claims data, registries) in research, clinical practice, and health policy rests in part on the assumption that RWD can accelerate clinical discovery at lower cost and greater efficiency than traditional randomized clinical trials [8]. RWD can support longitudinal studies, inform clinical decision‐making, and contribute to the generation of real‐world evidence (RWE, defined as the use, benefits, and evidence from analyses derived from RWD) [4, 9, 10, 11]. However, the use of RWD is contingent upon its quality and standardization. Challenges such as incomplete, inaccurate, or missing data, or bias and measurement errors can undermine the generation of RWE from RWD [12, 13, 14].

A primary traditional source of RWD is the EHR [14, 15]. However, an individual's health is shaped by a complex and interrelated set of factors that extend well beyond clinical data recorded in the EHR. These include care received across multiple health systems, lifestyle factors, environmental exposures, genetic information, and social and structural determinants of health [16]. The value of these data lies not solely in their scope, but in the ability to integrate interdependent data elements to better understand health trajectories and support more informed clinical, research, and public health decision‐making. Nontraditional sources of health relevant RWD, ranging from individual physiological monitoring [17] to social media [18], are rapidly emerging. The nontraditional RWD sources pose additional challenges to data quality and, ultimately, the generation of RWE [19].

## 2 The Scope of the Health Problem Addressed by SB‐PFT

Physical fitness is a measurable, exercise‐related attribute that reflects the resilience of integrated biological and behavioral systems. It is essential for growth, body composition, cognition, and learning, and is a robust predictor of health across the lifespan [20, 21, 22, 23]. Children are among the most spontaneously active humans [24, 25], yet fewer than 25% of US youth currently achieve the recommended 60 min of daily physical activity [26]. Low fitness in childhood is linked to obesity, metabolic disease, cardiovascular risk, behavioral disorders, and impaired immune function, with adverse effects persisting into adulthood [27, 28, 29, 30, 31, 32, 33]. Large trusted national organizations such as the American Heart Association note, “Development of a cost‐effective cardiorespiratory fitness measurement process that could readily be incorporated into office visits and in field settings to screen all youth periodically could help identify those at increased risk” [34].

Exercise‐associated respiratory symptoms and fatigue are commonly encountered in children and adolescents cared for by general pediatricians [35]. Reproducible assessments of physical fitness have been shown to be useful clinical biomarkers in elucidating underlying mechanisms of exercise‐associated respiratory symptoms and fatigue in children and adolescents [36, 37]. Thus, access to SB‐PFT results for individual children/adolescents and their care providers could benefit diagnosis and treatment.

## 3 Physical Fitness and Fit for Purpose: Pathways to Data Standards in the Challenging Conditions of School‐Site RWD

**FIGURE 1:**
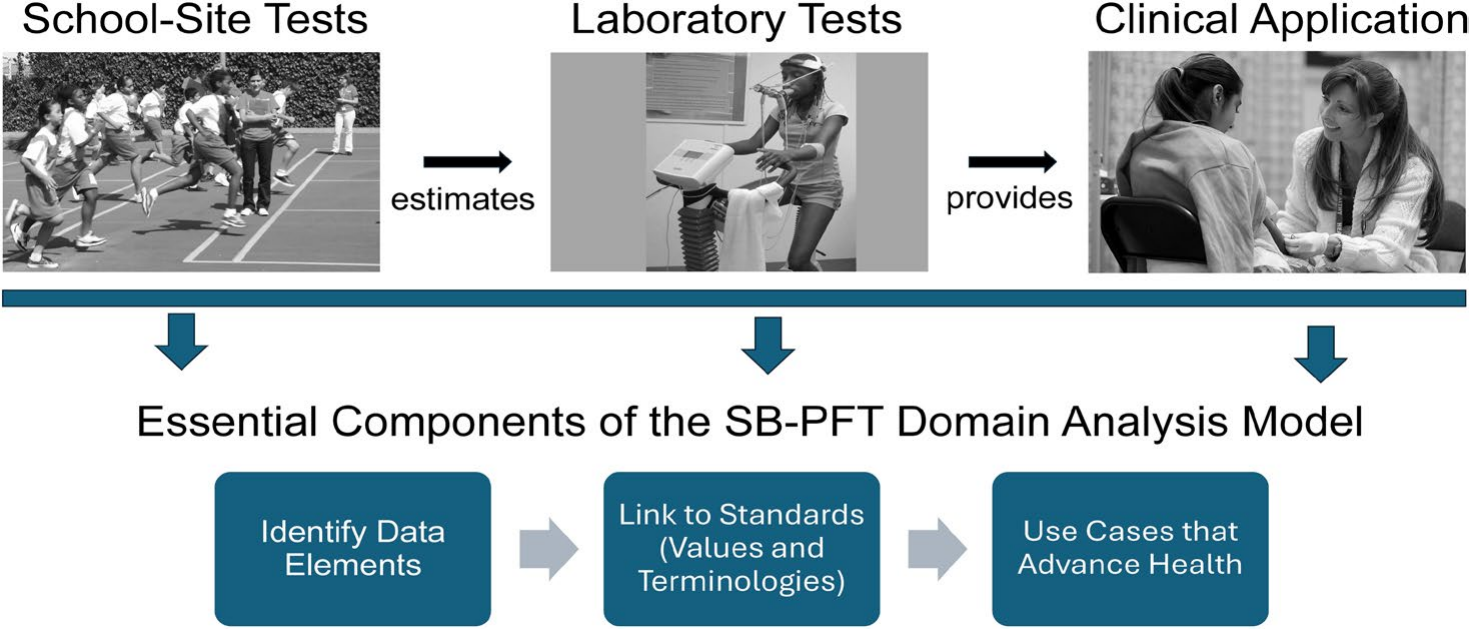
Challenges of school‐based physical fitness testing. SB‐PFT is based on the assumption that known, health relevant laboratory‐based physical fitness tests, such as the maximal oxygen uptake (V̇O_2_max) can be accurately estimated by field tests. The equipment and technological support required for laboratory testing render it unfeasible for the challenging environment of a school playground and classes often with 40 or more students and limited class time. The SB‐PFT DAM is a structured mechanism to embed data standards into school‐site testing and, in so doing, accelerate and improve the RWE generated from it to advance pediatric health at the individual and public health level.

As highlighted in Figure 1, SB‐PFT epitomizes the challenges inherent in capturing in real world settings those clinical biomarkers that are ideally collected in specialized laboratories. Because children spend most waking hours in school, physical education remains a critical venue for achieving healthy activity levels [38, 39, 40, 41]. School closures during the COVID‐19 pandemic sharply reduced youth fitness [42, 43, 44], while school‐based programs can effectively improve outcomes [45, 46]. School‐based physical fitness testing, pioneered through FitnessGram and its successors, has provided population‐level surveillance for decades [42, 47, 48, 49, 50].

Until recently, fitness testing was mandated annually within public schools in ~60% of US children and adolescents [51, 52]. Despite its reach, school‐site fitness testing suffers from inconsistent and sometimes insensitive implementation, outdated analytic methods, and limited pathways to deliver results to families and virtually nonexistent pathways to providers. Current inconsistencies threaten the continuation of school‐site fitness testing in states such as California [53]. We also recognize the potential for stigmatization and embarrassment in SB‐PFT is a legitimate concern, particularly among overweight students [54], and that care must and can be taken to avoid this. The last comprehensive National Academies review (2012) emphasized the urgent need for reevaluating fitness measures in youth and developing stronger links between field testing and health outcomes [47]. The review stimulated multiple recent efforts to address this knowledge gap [55], as well as the work described here to build an SB‐PFT DAM.

Attempts to embed school‐based data into the EHR must reconcile opportunities to expedite “real‐world data to practice” integration with necessary safeguards FERPA (Family Education Rights and Privacy Act) and HIPPA (Health Insurance Portability and Accountability Act) offer relevant populations [56, 57]. To that end, obtaining consent for data sharing with clinicians was included in our DAM model. In summary, we viewed the construct of the DAM as defined and implemented by HL7 as a key step in improving the quality of RWD collected in SB‐PFT across the United States.

## 4 Why we Built a Domain Analysis Model for School‐Based Physical Fitness Testing

The enormity of the health care crisis facing US children and the potential value of the unprecedented inclusion and scope of school‐site fitness testing led us to build a DAM with the goal of increasing data quality, rigor, and reproducibility. While EHR interoperability has advanced substantially through widespread adoption of standards (e.g., HL7, FHIR, LOINC, SNOMED CT, RxNorm), extending semantic and data interoperability to nontraditional real‐world data sources (e.g., wearables, patient‐generated health data, registries, social determinants of health, and cross‐sector public health datasets) remains challenging due to heterogeneous data generation contexts, inconsistent construct definitions, proprietary derivation algorithms, variable data quality/provenance, and the substantial effort required for mapping into common information models [19].

**FIGURE 2:**
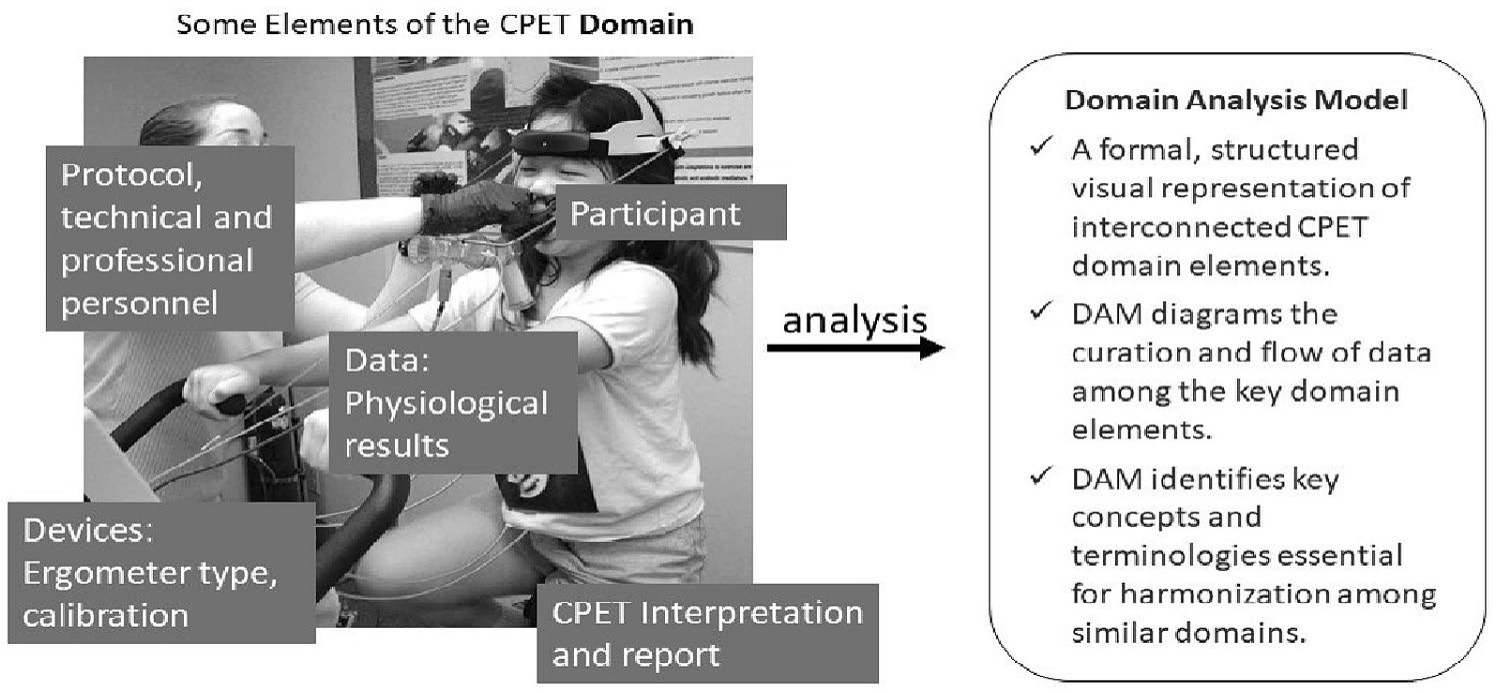
The THEMES DAM. The DAM was developed for both pediatric and adult CPET centers. The DAM identifies the essential elements of CPET and provides a structured graphical presentation and set of algorithms using UML. Building on the THEMES DAM, we developed a survey for pediatric CPET centers to gather data about concepts, terminologies, participants, devices, and analyses that can be used in the future to improve CPET quality and accelerate data sharing among different centers. The DAM was formally reviewed, critiqued, and vetted by the Clinical Interoperability Council of HL7 internationally.

The DAM seemed the most effective step in addressing threats to school‐site fitness testing and to specifically accelerate the inclusion of school‐site testing into the EHR. Our group had previous experience with DAM. In 2011, the UC Irvine Institute for Clinical and Translational Science (the NIH Clinical Translational Science Award, CTSA) convened an international panel of experts to address the clinical translational science gap in child health exercise research [58] and called for “disruptive innovation.” A major recommendation of the panel was to proceed with a formal DAM focused on laboratory‐based cardiopulmonary exercise testing (CPET) with the goal of creating semantic, syntactic, and data interoperability and harmonization. The resulting THEMES project, Terminology Harmonization in Exercise Medicine and Exercise Science [59, 60] was focused on cardiopulmonary exercise testing conducted in specialized human performance laboratories (Figure 2).

## 5 The Anatomy of a DAM (Table 1)

**TABLE 1:** Anatomy of a domain analysis model.

Essential activity	Objectives	Time and resource elements
Create panel of subject matter experts and key stakeholders	Convene individuals with content and experiential knowledge of the domain. Include academic, clinical, and community representatives	Costs include proper remuneration for subject experts and community stakeholders time
Activity model Use case model Process flow model	Documents the dynamic or process flow aspect of the domain.	Requires expertise in Unified Modeling Language and data and terminology harmonization.
Information model Subject areas and classes Definitions and permitted values	Documents the information content aspect of the exercise medicine domain. Includes the set of classes, class relationships, and attributes within the scope of the DAM Provides context for inclusion and definition of data elements.	Often, subject matter experts and community stakeholders have narrow and case‐specific views on definitions and normative values. Consensus‐building and inclusive leadership is needed to develop common models
Data elements	Data items identified by the subject matter experts and essential for the domain. Grouped by class. Definition is provided for each data element as well as definitions for each class and data element permitted value.	Technical skills and knowledge in the use of existing data classification appropriate for fit‐for‐purpose aspects of the DAM are essential.
Class diagram	Formally defines the meaning, structure, and constraints of a domain so that humans and machines can interpret data consistently—independent of any particular software implementation.	The correct combination of data standards and UML expertise along with judicious project leadership is the most reliable pathway to successful completion of a DAM.
Administrative support	Convene meetings and schedules Curate DAM documents Minutes, progress notes, evaluation	The UC Irvine Institute for Clinical and Translational Science (NIH CTSA) supported a part time coordinator. This was seen as contributing to the NCATS mission to advance the science of translation.

The formal concept of the DAM originated from collaborations between health care providers and information scientists working to improve the efficiency and harmonization of complex health care activities (personal communication, W. Ed Hammond, PhD, Duke University) [61, 62]. DAMs offer a methodological approach to achieving interoperability and data harmonization by ensuring data quality across domains. DAMs can provide both a dynamic (business processes) and static (conceptual) representation of a particular domain and establish a standard framework to allow for standardized communication across disparate systems.

The Life Sciences Domain Analysis Model (LS DAM) [63], for example, facilitated static harmonization across translational research domains by integrating concepts from molecular biology, clinical trials, and specimen management into a unified framework. DAMs lead to standards‐based interoperability consisting of common data models and computable common data elements (CDEs), all foundational in facilitating information exchange between entities. CDEs provide a standardized semantic representation of data through defined question text, precise definitions, and permissible response values, enabling consistent interpretation and reuse of data across systems and contexts. The DAM approach supports high‐quality data integration, scalable analytics, and reproducible research, which are essential for generating reliable RWE.

## 6 Key Building Blocks of the SB‐PFT DAM

The SB‐PFT DAM aims to address existing gaps in data quality and provide avenues for interoperability by enabling standardized data collection and exchange across: (1) individual student, parent, and family; (2) local and regional school; (3) departments of health and policy entities; (4) associated clinical providers; and (5) research settings. By capturing both the behavioral and informational aspects of SB‐PFT, the SB‐PFT DAM provides a foundation for consensus building, standardization, and cross‐domain integration. It supports the integration of SB‐PFT data with EHRs to improve pediatric health outcomes, facilitates personalized health interventions, and enables the use of physical fitness data in public health research and surveillance.

### 6.1 The SB‐PFT Working Group (Table 2)

**TABLE 2:** SB‐PFT DAM working group.

Subject matter expertise	Working group member
Clinical and provider expertise	Pediatric pulmonologist General pediatrician School Nurse Specialist
School administrators and physical education instructors	School principal and Physical Education Consultant School District Director of Family and Community Engagement School District Coordinator of Physical Education
Stakeholder perspective	Current student‐parent dyad in regional school
Health policy officials	Chief Medical Officer, Regional Health Care Agency
Academic/researcher	Professor of Pediatrics, (specialist in exercise physiology in growth and development) Professor of Kinesiology (specialist in school based fitness testing) Professor, Informatics Science Graduate Student
DAM process	Unified Modeling Language Data Standards development
Administrative support	Project coordinator

Modeled after the collaborative approach in cardiology, oncology, and mental health associated with the long COVID Multiple Chronic Conditions Electronic Care Plan project [64], a panel of subject matter experts and community partners was established to identify and define data elements related to SB‐PFT. This panel included individuals from diverse backgrounds and perspectives and consisted of experts in the areas of clinical medicine, physical education, public health, nursing, informatics, education, physical fitness research, kinesiology, and standards development. Parents of students who have or will undergo the SB‐PFT were also included. The panel was led by an exercise physician expert, a standards expert, and supported by HL7 modelers.

### 6.2 Data Element Identification

The process of identifying appropriate data elements related to SB‐PFT followed an iterative approach. The concepts were gathered through an extensive literature review, systematically cataloged in a spreadsheet, and categorized by data elements including their multiple synonyms and various definitions. Through regular discussions, additional data elements and definitions were recommended to be included based on expert opinion and authoritative sources.

Each data element incorporated into the SB‐PFT DAM was established through a community structured consensus decision‐making process designed to ensure methodological rigor, stakeholder alignment, and semantic clarity. Monthly convenings served as the primary forum for deliberation, during which each proposed data element was subjected to comprehensive scrutiny. During these sessions, participants engaged in a systematic review and discussion of each element's relevance and definitional precision. Stakeholders were encouraged to contribute domain‐specific insights, raise concerns regarding ambiguity or redundancy, and propose refinements to improve semantic interoperability and practical utility. The deliberative process emphasized transparency and inclusivity, with decisions reached through iterative rounds of feedback.

Final agreement on each data element required a simple majority agreement. This ensured that the resulting data definitions were not only technically sound but also reflective of the diverse operational realities across educational, clinical, and research settings. The consensus process served as a foundational mechanism for establishing a robust, interoperable, and stakeholder‐endorsed data architecture for school‐based physical fitness testing.

### 6.3 Partnership With Health Level 7 (HL7)

A formal project was established with the HL7 Patient Care (PC) Work Group to advance the specification of standards necessary for the structured capture, exchange, and utilization of SB‐PFT data and ensure interoperability across educational, research, and health systems. Health Level Seven is an American National Standards Institute (ANSI) accredited standards development organization that has been creating health care standards for decades [65, 66, 67]. Working with the HL7 PC Work Group, our team refined the dynamic (behavioral) and static (information) representations of SB‐PFT activities, ensuring that the model accurately captured the workflows, actors (entities), and data exchanges inherent to physical fitness testing in school settings. These representations were formalized using Unified Modeling Language (UML) [68] and structured into use case diagrams, activity flows, and class models that delineate the relationships among participants, devices, observations, and reporting entities.

The partnership also facilitated the identification and harmonization of terminology and value sets, drawing from authoritative sources such as LOINC and SNOMED. This ensured that the SB‐PFT data elements could be meaningfully integrated into EHR systems and leveraged for clinical decision support, care coordination, and longitudinal health monitoring. By embedding SB‐PFT data within the broader HL7 ecosystem, the model enables pediatric healthcare providers to access and interpret physical fitness data in the context of routine care, thereby enhancing opportunities for family engagement, personalized health interventions, and early identification of risk factors associated with poor cardiorespiratory fitness and other health‐related outcomes. Collaboration with the HL7 PC Work Group transformed the SB‐PFT DAM from a domain‐specific conceptual framework into a standards‐based, interoperable model capable of supporting real‐world data exchange and multi‐domain integration.

## 7 SB‐PFT DAM Products

### 7.1 SB‐PFT Data Elements and Structure

The SB‐PFT working group decided on the SB‐PFT data elements, after consensus and extensive discussions. Six overarching fitness categories were defined: aerobic capacity, body composition, muscle strength, muscle endurance, muscle power, and muscle flexibility. Physical activity data elements were assigned to a fitness category according to current practices and expert consensus. Each element was mapped to standard terminologies where available, including LOINC codes for test results (e.g., 66210‐6 for mile walk completion, 66212‐2 for push‐up count), SNOMED CT for categorical responses (e.g., Yes/No/Unknown), and NIH Common Data Elements (CDEs) for consent and assent (Table 3).

**TABLE 3:** Assignment of LOINC codes to several SB‐PFT data elements.

Category	Field/question name	Data element	Code
Aerobic capacity	One‐mile walk: participated?	Mile walk–participation	62814‐9
Body composition	Participant's weight	Weight	29463‐7
Muscle strength	Number of push‐ups completed	Push up number–completion	66212‐2
Muscle flexibility	Right shoulder stretch: arm	Shoulder stretch–right arm	66243‐7

The value sets developed for SB‐PFT were designed to be broad and inclusive, ensuring compatibility with diverse data collection practices across school districts while maintaining alignment with clinical and research standards. Where standardized codes were unavailable, elements were informed based on existing data elements and vocabularies (e.g., FitnessGram, PhenX, CDC NYFS). This approach maximized the likelihood of capturing relevant data in a format familiar to both educators and healthcare providers. Given the variability in physical fitness testing protocols and the absence of universally accepted performance thresholds for youth fitness, the working group prioritized the inclusion of data elements that reflect observable, measurable outcomes (e.g., time to complete a one‐mile run, number of push‐ups completed) rather than normative interpretations. This decision supports the use of SB‐PFT data in longitudinal research, personalized health interventions, and integration with EHRs for clinical decision support.

**FIGURE 3:**
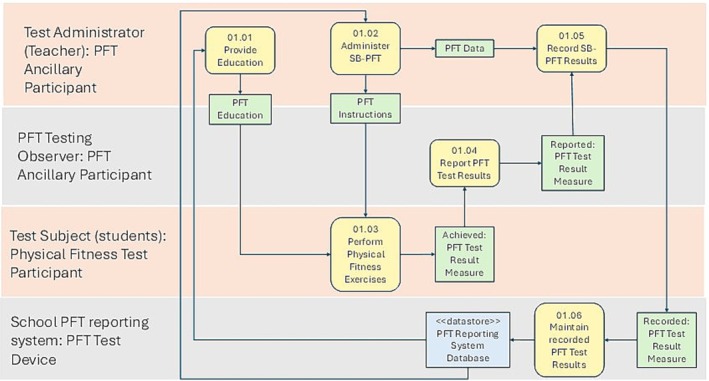
School fitness Domain Analysis Model. Shown here is the overall data flow in the Unified Modeling Language format as accepted by HL7. The DAM provides interoperability for data flow and standards for terminology positioning school physical fitness tests (PFT) for eventual incorporation in the electronic health record. The numbers in the figure are Unified Modeling Language cardinalities and define the allowable number of occurrences of a data element or relationship—whether something is optional or required, and whether it can occur once or multiple times.

Additionally, the use of UML enabled the project team to visually and semantically articulate the complex workflows, actors, and data exchanges inherent to SB‐PFT. The team constructed a series of UML artifacts, including use‐case diagrams, activity process flows, and class diagrams, that captured the full data lifecycle of SB‐PFT, from test administration and data collection to multi‐stakeholder reporting and integration with EHRs (Figure 3).

The UML‐based behavioral viewpoint delineated the sequence of activities involved in SB‐PFT, such as educator‐led instruction, student participation in physical fitness exercises, and the recording and reporting of test results. Swimlane diagrams were employed to clarify the roles and responsibilities of each actor (e.g., student, teacher, observer, administrator) and to map the flow of information across systems. Concurrently, the information viewpoint was developed to define the structure and semantics of the data elements, organized into six subject areas: Physical Fitness Test Participant, Physical Fitness Test, PFT Ancillary Participant, Physical Fitness Test Device, Physical Fitness Report, and PFT Report Recipient.

### 7.2 SB‐PFT Use‐Cases and Story Lines (Figure 4)

**FIGURE 4:**
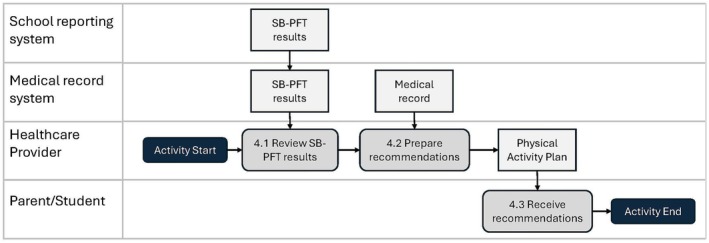
Story line use case for SB‐PFT DAM. This UML representation illustrates the flow of data from school‐site to patient interaction if SB‐PFT data can be successfully included in a student's EHR.

A powerful product of the DAM is use‐case story lines which integrate UML data visualization with likely scenarios of data exchange in real world settings. Here is an example of a use‐case storyline focused on SB‐PFT Reporting to a Healthcare Provider.

*Current state*: There are no known large‐scale systems in place for sharing school based physical fitness testing results with healthcare providers or integrating them into electronic health record systems.

*Challenges*: Integrating physical fitness data with electronic health records to provide healthcare providers a more complete view of the student's health. This should enable personalized health advice and proactive management of the student's physical development.

*Participating actors*: Healthcare provider, Parent, Student.

*Story scenario*: Scott completed his physical fitness test at school several weeks ago. Today, he's going to his pediatrician with his parents for an annual physical exam. Dr. Smith has been Scott's pediatrician for the past several years. Scott's parents provided their consent after the physical fitness test, allowing the school to share the SBPFT data with his physician. As she reviews Scott's chart before seeing him in the exam room, Dr. Smith notices his SB‐PFT results in the EHR, along with other relevant health information such as height, weight, maturational status, sleep patterns, and hospitalizations.

After reviewing this information, Dr. Smith interviews Scott and his parents about the past year. Following the physical exam and considering the results from the fitness tests and Scott's health history, Dr. Smith prepares a tailored physical activity plan. The plan is aligned realistically to environmental factors, availability and access to play and organized programs of physical activity at school and other community venues for Scott and his peers, and cost. She then discusses this plan with Scott and his parents to ensure it aligns with Scott's health needs and lifestyle.

*Review PFT results*: The healthcare provider reviews the PFT results in the electronic medical record.

*Prepare recommendations*: The health care providers will evaluate the PFT results with the patient's health history, other health markers, and relevant patient health history to determine and prepare tailored physical activity recommendations.

*Receive recommendations*: The parent and student will discuss the physical activity recommendations with the healthcare provider.

## 8 Scalable Implications of the SB‐PFT DAM

The development of the SB‐PFT DAM represents a foundational advancement in the transformation of field‐collected physical fitness data into structured, interoperable RWD. By aligning with HL7 standards, the SB‐PFT DAM addresses long‐standing challenges in the collection, interpretation, and reuse of physical fitness data collected in educational settings. The SB‐PFT DAM is designed to resolve several critical semantic and structural barriers that have limited the utility of SB‐PFT data. First, terminology variation across school districts can lead to inconsistent labeling of fitness constructs, which introduces ambiguity in data interpretation and hinders cross‐site comparability. The DAM mitigates this issue by establishing harmonized vocabulary, drawing from authoritative publications [69], Institute of Medicine (IOM), and the Centers for Disease Control and Prevention (CDC), and binding data elements to standard terminologies including LOINC, SNOMED CT, and NIH CDEs. Second, inconsistency across schools can pose a significant challenge to data reliability. Variations in test administration procedures, such as differences in scoring methods, can introduce measurement bias and reduce the comparability of results. The SB‐PFT DAM addresses this by defining protocol attributes and supporting mappings to reference procedures, enabling standardized documentation of test conditions and facilitating normalization across sites.

To enhance their usability, the data standards or CDEs should be computable and encoded with technical formatting such as defined data structures, data dictionaries, and data modeling languages to generate outputs that can auto‐generate case report forms in electronic data capture forms such as REDCap. The DAM explicitly models these representational attributes, ensuring that each data element is accompanied by clear semantic and syntactic constraints. This includes the use of controlled vocabularies for categorical responses, quantitative ranges, and unit standards. Finally, limited interoperability can prevent SB‐PFT data from being linked to EHRs, public health databases, or research networks. The SB‐PFT DAM serves as a foundational data exchange, enabling seamless integration with clinical and research systems.

## 9 Lessons Learned

The SB‐PFT DAM initiative yielded several important insights that may inform future efforts in health data standardization:

### 9.1 Stakeholder Engagement

The success of the DAM was predicated on sustained collaboration among a diverse group of stakeholders, including educators, clinicians, informaticians, standards development experts, and public health officials. This multidisciplinary engagement was essential for ensuring that the model reflected real‐world practices and met the needs of all user communities.

### 9.2 Iterative Refinement

The modeling process was inherently iterative. Feedback loops enabled through structured review forms, monthly meetings, and asynchronous commentary were critical for refining data definitions, resolving ambiguities, and improving model usability.

*Scalability and adaptability*: The DAM was designed with scalability in mind. Its modular structure supports a wide range of use cases, from local school wellness programs to national surveillance initiatives. The model's adaptability also allows for future extensions, such as the inclusion of new fitness modalities.

### 9.3 Limitations

Despite its strengths, the SB‐PFT DAM has several limitations. First, while the model includes metadata fields for device calibration, it does not directly address the operational gaps in calibration practices, which may compromise data accuracy. Second, although the DAM supports protocol standardization, it does not enforce uniform testing procedures across schools, which may continue to introduce variability in data quality. Third, the model's reliance on existing terminologies is constrained by the availability of atomic codes for certain fitness activities; in such cases, broader panels or proxy codes were used, which may limit granularity. Finally, implementation of the DAM in real‐world settings will require investment in training, infrastructure, and policy alignment, factors beyond the scope of the DAM.

## 10 From DAM to FHIR and Learning Health System (Figure 5)

**FIGURE 5:**
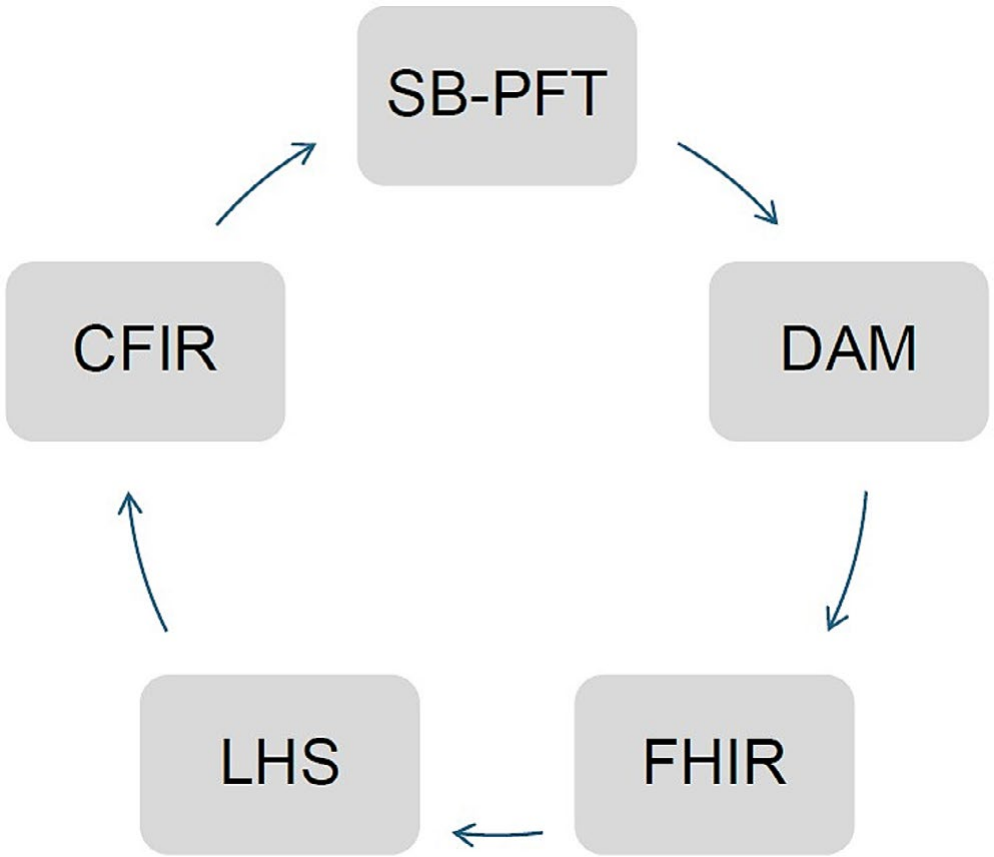
Scaling/generalizability of SB‐PFT to improve pediatric health. The Domain Analysis Model (DAM) will create common data elements, standardization, and terminology harmonization as outlined in the text. Fast Healthcare Interoperability Resources (FHIR) will accelerate the inclusion of SB‐PFT into the electronic health record. While a DAM itself does not create a true learning health system, the DAM work products can facilitate the development of a learning health system (LHS). Further refinement can employ modern principles of implementation science (Consolidated Framework for Implementation Research, CFIR) for a continuous improvement cycle of school‐site testing.

The DAM provides a framework for implementation. We propose two distinct pathways to achieve the longer‐term goals of the SB‐PFT DAM, namely, inclusion of fitness data into the EHR and scaling of the DAM defined standards at scale.

### 10.1 From DAM to FHIR‐Enabled EHR Integration

Fast Healthcare Interoperability Resources (FHIR) is assuming an increasing role in enhancing the interoperability of healthcare information systems [70]. The SB‐PFT DAM provides a standards‐based bridge between SB‐PFT and clinical information systems by establishing a clear semantic and structural foundation for FHIR implementation. By explicitly modeling participants, fitness tests, observations, devices, roles, and reporting workflows, the DAM enables SB‐PFT data to be mapped to existing FHIR resources (e.g., Patient, Observation, Procedure, Device, Diagnostic Report) as computable, interoperable health data rather than unstructured external reports. This approach preserves measurement context, provenance, and timing while allowing fitness data collected in educational settings to be incorporated into longitudinal EHR records alongside traditional clinical data. The DAM thus shifts school‐based fitness testing from a disconnected surveillance activity to a clinically interpretable data source that can support pediatric preventive care, clinical decision‐making, and research reuse.

### 10.2 Scaling SB‐PFT Standards Through a Learning Health System (LHS)

Beyond technical interoperability, the SB‐PFT DAM establishes the prerequisites for embedding school‐based fitness testing within an LHS that supports continuous learning and improvement. By separating domain semantics from local implementation, the DAM allows standardized data elements, workflows, and reporting structures to be reused, evaluated, and refined across diverse school districts, health systems, and research contexts. When SB‐PFT data are exchanged through interoperable standards and linked with clinical, public health, and contextual data, they can be analyzed at scale and fed back to educators, clinicians, families, and policymakers to inform practice and policy. This iterative cycle, data to knowledge, knowledge to action, supports generalizability, equity‐focused evaluation, and sustainable scaling of SB‐PFT as a nontraditional but high‐value source of real‐world data within pediatric population health.

## 11 Conclusion

The SB‐PFT DAM represents a critical step toward unlocking the potential of SB‐PFT as RWD. The current DAM gained initial approval by the HL7 Patient Care Work Group and our SB‐PFT group's response to comments made by HL7 members. HL7 official publication will occur when the review process is complete. By addressing key semantic and structural challenges and aligning with HL7 and FHIR standards, the model enables interoperability, comparability, and reuse across educational, clinical, and research domains. While challenges remain in implementation and calibration, the DAM builds the infrastructure for the creation of an SB‐PFT LHS. Collectively, these activities create a robust foundation for future work in pediatric health surveillance, personalized care, and public health policy. The experience presented here can serve as a roadmap for addressing critical issues of data standardization and RWE generation in nontraditional instances of RWD.

## Funding

This work was supported by National Center for Advancing Translational Sciences (TR002004 and TR004927).

## Conflicts of Interest

The authors declare no conflicts of interest.

## Data Availability

The data that support the findings of this study are available on request from the corresponding author. The data are not publicly available due to privacy or ethical restrictions.
